# CHANGE IN ENGAGEMENT WITH PRODUCTIVE SOCIAL ACTIVITIES: HOW IT PREDICTS LATE-LIFE DEPRESSION OVER TIME

**DOI:** 10.1093/geroni/igad104.2144

**Published:** 2023-12-21

## Abstract

Research has identified that engaging in activities is associated with lower levels of depression among older adults. However, there is less discussion about how changes in participation in different types of productive social activities (PSAs) affect depression in later life. This study examined data from Taiwan Longitudinal Study on Aging, including 2,865 participants aged 55 or above, from 2011 (T1) to 2015 (T2). The relationship between changes in PSAs and depression was tested through six regression models. Results indicated that: 1. Older adults who dropped out from work or were continuously unemployed experienced higher levels of T2 depression compared to those who remained continuously employed. 2. Older adults who never volunteered experienced higher levels of T2 depression compared to those who continuously engaged in volunteering. 3. Engaging in informal caregiving for relatives did not reduce T2 depression. In contrast, older adults who dropped out of providing care for other adults experienced lower levels of T2 depression compared to those who continuously offered care. This study shows that older people tend to gradually decrease their engagement in PSAs in later life, but those who continue to engage in PSAs tend to have lower levels of depression over time. Employment and volunteering appear to predict depression. It is worth noting that while informal caregiving is also considered a PSA, people who stopped providing care for other adults experience lower levels of depression. This research calls for further investigation into mechanisms of varied type of PSAs affect wellbeing in later life.

